# Diffuse sclerosing papillary thyroid carcinoma in a child

**DOI:** 10.1007/s00247-026-06691-2

**Published:** 2026-06-13

**Authors:** A. Yigitcan Tacyıldız, H. Nursun Ozcan

**Affiliations:** Hacettepe University School of Medicine, Sıhhiye, Ankara, 06100 Turkey

An otherwise healthy 11-year-old girl presented with painless neck swelling. Transverse ultrasonography of the neck (**a**) shows diffuse milimetric hyperechogenic foci in both thyroid lobes. The left lobe was nearly filled by a solid, hypoechoic nodule with coarse calcifications (arrow). A solid, hypoechoic nodule was also seen in the isthmus. Bilateral cervical metastatic lymph nodes were present, with the largest measuring 21 mm in short-axis diameter. Findings were consistent with the diffuse-sclerosing variant of papillary thyroid carcinoma, an infrequently encountered but aggressive subtype more commonly seen in younger patients. Fine-needle aspiration biopsy of the nodule confirmed the diagnosis. A systematic evaluation for metastatic disease was done. Coronal maximum intensity projection (MIP) computed tomography image of the chest in lung windows (**b**) shows diffuse metastatic nodules throughout both lungs. The patient underwent total thyroidectomy, modified radical neck dissection followed by radioactive iodine treatment. She continues thyroid suppression therapy with regular surveillance.

**Figure:**
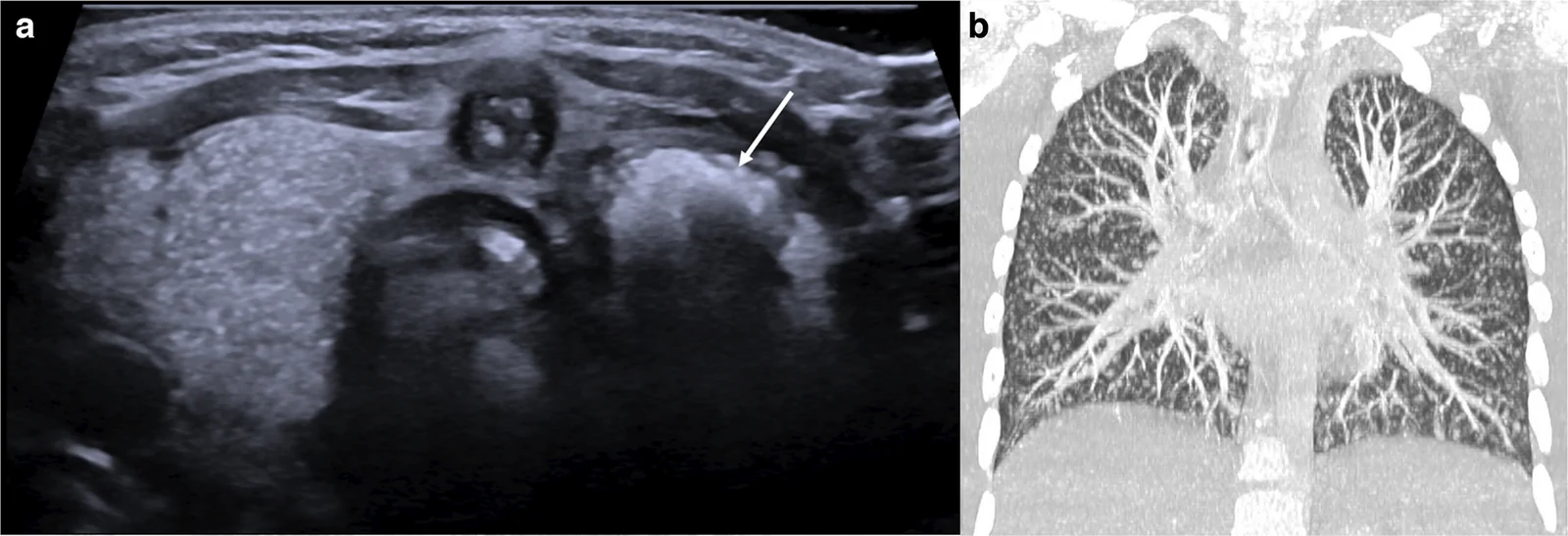

## Data Availability

No datasets were generated or analysed during the current study.

